# Flexibility of EF-hand motifs: structural and thermodynamic studies of Calcium Binding Protein-1 from *Entamoeba histolytica* with Pb^2+^, Ba^2+^, and Sr^2+^

**DOI:** 10.1186/2046-1682-5-15

**Published:** 2012-08-20

**Authors:** Shivesh Kumar, Ejaz Ahmad, Sanjeev Kumar, Rizwan Hasan Khan, Samudrala Gourinath

**Affiliations:** 1 School of Life Sciences, Jawaharlal Nehru University, New Delhi, India; 2 Present address: Department of Biological Sciences, Purdue University, West Lafayette, IN, 47907, USA; 3 Interdisciplinary Biotechnology Unit, Aligarh Muslim University, Aligarh, India; 4 Department of Biochemistry, Jamia Hamdard University, New Delhi, India

**Keywords:** Calcium sensor, Calcium binding protein, Coordination geometry, EF-hand motifs, Anthropogenic toxicant, Domain swapped manner, Anomalous signal

## Abstract

**Background:**

EF-hand proteins can be activated by the binding of various heavy metals other than calcium, and such complexes can disturb the calcium-signaling pathway and cause toxicity and disease causing state. So far, no comprehensive study has been done to understand different heavy metals binding to calcium signaling proteins.

**Results:**

In this work, the flexibility of the EF-hand motifs are examined by crystallographic and thermodynamic studies of binding of Pb^2+^, Ba^2+^ and Sr^2+^ to Calcium Binding Protein-1 from *Entamoeba histolytica* (EhCaBP1). The structures of the EhCaBP1- heavy metal complexes are found to be overall similar, nevertheless specific differences in metal coordination, and small differences in the coordination distances between the metal and the ligands in the metal binding loop. The largest such distances occur for the Ba^2+^- EhCaBP1 complex, where two bariums are bound with partial occupancy at the EF2 motif. Thermodynamic studies confirm that EhCaBP1 has five binding sites for Ba^2+^ compared to four binding sites for the other metals. These structures and thermodynamic studies reveal that the EF-hand motifs can accommodate several heavy atoms with similar binding affinities. The binding of Ca^2+^ to the 1^st^, 2^nd^ and 4^th^ sites and the binding of Ba^2+^ to the 1^st^, 2^nd^, 4^th^ and 5^th^ sites are both enthalpically and entropically driven, whereas the binding of Sr^2+^ to the 1^st^, 2^nd^ and 4^th^ sites are simply enthalpy driven, interestingly in agreement with ITC data, Sr^2+^ do not coordinate with water in this structure. For all the metals, binding to the 3^rd^ site is only entropy driven.

**Conclusion:**

Energetically, Ca^2+^ is preferred in three sites, while in one site Ba^2+^ has better binding energy. The Sr^2+^-coordination in the EF hand motifs is similar to that of the native Ca^2+^ bound structure, except for the lack of water coordination. Sr^2+^ coordination seems to be a pre-formed in nature since all seven coordinating atoms are from the protein itself, which also correlates with entropy contributions in Sr^2+^ binding. These findings improve our understanding of metal association with calcium binding proteins and of metal induced conformational changes.

## Background

The presence of toxic heavy metals in the environment is of particular concern since they can accumulate in the body and cause significant health problems even at low concentrations [1,2]. Although the coordination geometries of many proteins are well suited for binding essential metal ions, they can also often interact with a number of heavy metals. Pb^2+^ is the most studied and prominent such toxicant: it displaces both Ca^2+^ and Zn^2+^ in proteins, affects neurotransmitter release and neuronal growth, and causes anemia, kidney damage, hypertension as well as male infertility [3-5] At the molecular level, Pb^2+^ has been shown to specifically target voltage-gated calcium channels [6], skeletal muscle troponin C (TnC) [7] and can also activate calmodulin (CaM) at low concentration [8].

Cationic lead is able to substitute for Ca^2+^ in the regulation of CaM’s function [9,10]. The activity levels of many target proteins are indeed affected by Pb-CaM as they are by Ca-CaM [11], leading to abnormal responses. Other metal ions such as Mg^2+^, Ba^2+^, Sr^2+^, Hg^2+^, Cd^2+^ and most lanthanides also show affinity with natural and engineered calcium binding proteins (CaBPs) including CaM [12-15]. However, the maximum activation decreases in the order Pb^2+^ > Ca^2+^ > Sr^2+^ > Ba^2+^ > Cd^2+^ as demonstrated previously in the EF-hand motif of D-galactose binding protein [16]. Moreover, the activity of downstream CaM-mediated functions depends on the concentration of the metal ion.

Structural and binding affinity investigations of calcium binding proteins aimed at understanding the flexibility and plasticity of EF-hand motifs, as well as understanding the structures of their interactions with various heavy metal ions, are very limited. Among the EF-hand containing proteins, the structure of CaM has been determined in complex with couple of heavy metals: one case with Pb^2+^[17,18] and another with partly bound Ba^2+^, in which only EF-hand motif 2 was bound with the ion [17].

**Figure 1:**
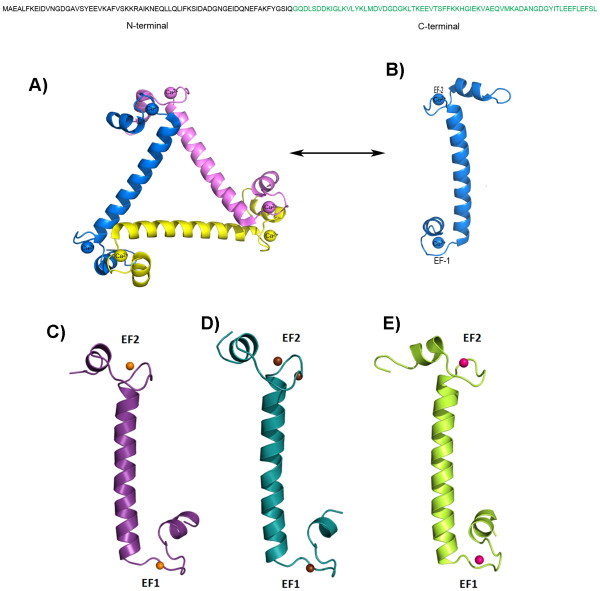
**Crystal structure of EhCaBP1.** The sequence on the top shown with the N and C-terminal domains. In the structure only the N-terminal domain was traced since the C-terminal domain is disordered. **A**) The three molecules of the N-terminal domain come together to form a trimer. **B**) Each monomer is an extended structure with two EF hand motifs separated by a long helix. This calcium bound structure was reported earlier by Kumar et al., 2007. **C**-**E**) The heavy atom complex structures. **C**) Sr^2+^ complex, **D**) Ba^2+^ complex and **E**) Pb^2+^ complex of EhCaBP1 have overall similar structures to the native Ca^2+^ bound structure.

So far, detailed comparative binding studies of different heavy atoms to any single CaBP, including CaM, have not been carried out. Here we have carried out such studies in detail with EhCaBP1*,* which is a calcium binding protein from *Entamoeba histolytica*. EhCaBP1 has four EF-hand motifs, each of them able to bind one calcium ion similar to that of calmodulin. The crystal structure of EhCaBP1 has been reported previously at 2.4 Å resolution by our group, where only the N-terminal half was traced and the C-terminal domain was found to be missing due to the presence of the flexible linker region between these two domains [19]. The overall structure is trimeric in which EF-hand 1 of one molecule interacts with EF-hand 2 of the other molecule in a domain swapped manner to form an assembled domain similar to that of the calmodulin N-terminal domain (Figure 1). The trimeric and monomeric forms of EhCaBP1 are in equilibrium with each other depending on the solvent conditions [20].

In this study, we investigate the specificity and flexibility of EF-hand motifs of EhCaBP1 in detail, using three heavy atoms: Pb^2+^, Ba^2+^ and Sr^2+^. We have successfully replaced the calcium ions in EhCaBP1 with each of these heavy atoms and have determined their structures. The association profile of Ca^2+^, Sr^2+^ and Ba^2+^ to EhCaBP1 has been determined by isothermal titration calorimetry (ITC) at physiological pH. Sr^2+^ and Ba^2+^ have similar binding affinities but a little lower binding energy (ΔG) compared to Ca^2+^ for the first, third, and fourth sites, while for the second site, Sr^2+^ and Ba^2+^ have better binding affinities. The heavy metal complex structures are similar to the native structure, with minor differences in the EF-hand motifs. There are small differences in the coordination distances between the metals and the ligands; the distances are largest for EhCaBP1-Ba. These results help explain the physiological basis for toxicity of these heavy metals by providing structural and biochemical insights into how these metal ions can effectively substitute for Ca^2+^ in a molecule that is central to several regulatory processes.

## Results

### Lead complex

**Figure 2:**
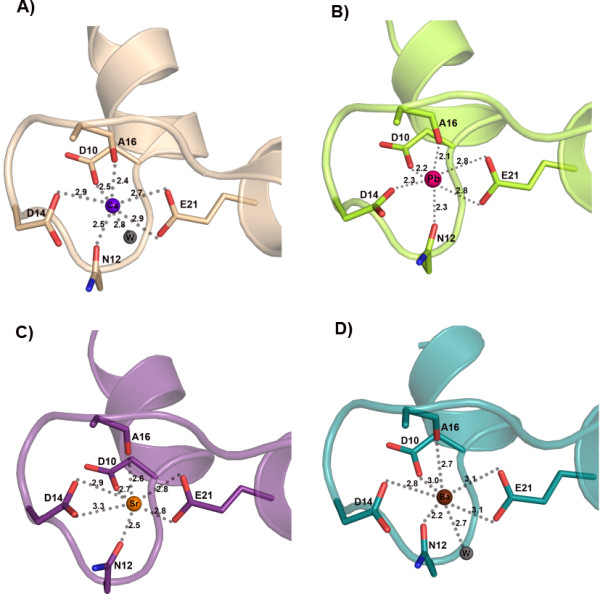
**EF-hand motif 1 of EhCaBP1 showing the heavy-metal coordination.** (**A**) Calcium coordination in the calcium binding loop-1 of EhCaBP1. Calcium coordinates with seven of its ligands including one water molecule to satisfy the coordination geometry. The coordination distances are shown. (**B**) Lead coordination in the calcium binding loop-1 of EhCaBP1. Lead co-ordinates with six ligands in the EF-hand motifs. The coordination distances between the Pb^2+^ and ligand oxygen atoms for EF-hand 1 motif range between 2.1 Ǻ and 2.8 Ǻ. (**C**) Strontium coordination in the calcium binding loop-1 of EhCaBP1. Strontium co-ordinates with seven ligands in the EF-hand motif. The coordination of Sr^2+^ is similar to that of Ca^2+^ coordination except for the absence of water molecule in the coordination. The 3^rd^ aspartate residue is donating both of its oxygen ligands to satisfy the coordination geometry. The coordination distances between the ion and oxygen atom for the EF-hand 1 motif range between 2.1 Ǻ and 2.8 Ǻ. (**D**) Barium coordination in the calcium binding loop-1 of EhCaBP1. Barium coordinates with seven ligands including one water molecule to satisfy the coordination geometry. The coordination distances are shown. The coordination of Ba^2+^ is similar to that of Ca^2+^. The coordination distances between the ion and oxygen atom for EF-hand 1 motif range between 2.4 Ǻ and 2.9 Ǻ.

In calmodulin (CaM), Pb^2+^ has been reported to exhibit higher affinity towards the calcium binding site than Ca^2+^ itself, and induce the protein into a constitutively active state [8,11]. The calcium ions were indeed replaced by lead ions, in both EF hands, when EhCaBP1 was co-crystallized with Pb^2+^, as indicated by the anomalous signal (Table 1 and see below), peaks in difference fourier maps, and improved crystallographic refinement upon including the heavy metal. As is found in the native, Ca^2+^-bound EhCaBP1 structure, the EF-hand motifs are also connected by a long helix in the Pb^2+^-bound EhCaBP1 structure. Since Pb^2+^ has one extra lone pair of electrons in the outermost shell, in contrast to Ca^2+^, Pb^2+^ generally interacts with six ligands, in contrast to Ca^2+^, which interacts with seven ligands. In the complex structures, Pb^2+^ thus cannot interact with the additional water molecule that Ca^2+^ interacts with. In the EhCaBP1-Pb^2+^ complex structure, six oxygens from the both EF hand motifs are interacting with Pb^2+^ (Figure 2B) as expected. The average coordination distances between Pb^2+^ and its ligands are a little lower than between Ca^2+^ and its ligands in the EF1 motif, but they are almost the same in the EF2 motif. Note that the Ca^2+^ and Pb^2+^ coordination distances were shown to be the same in the Calmodulin complex structures [17].

**Table 1:** Anomalous signal evaluation

**Data set**	**CaBP1-Ba**	**CaBP1-Pb**
Wavelength (Å)	1.54178	1.54178
Anomalous f”	9.025	8.934
Anomalous signal	0.1071	0.0622
Bijvoet pairs	4892	7281
Lone Bijvoet mates	187	24
**Expected Anomalous signal**		
**AutoSol**		
No. of Heavy atom sites	6	4
FOM	0.40	0.18
CC	0.67	0.41
Residues built	91	75
R/Free_R (%)	0.41/0.48	53.0/56.0
**AutoBuild**		
Residues built (%)	80	65
Map CC	0.72	0.64
R/Free_R	0.42/0.47	47.0/55.0

### Strontium complex

The affinity of Sr^2+^ for CaM is lower than that of Ca^2+^ and Pb^2+^, but higher than that of Ba^2+^. Moreover, strontium and calcium have comparable cation sizes and hydration energies [10]. The overall structures of the Sr^2+^ bound and Ca^2+^ bound EhCaBP1 complexes are similar to each other, except for the metal coordination. A water molecule was not found to be coordinating Sr^2+^, as is observed in the Ca^2+^ bound as well as Ba^2+^ bound structures (Figure 2C). Both oxygens of the 3^rd^ aspartate residue (residue 14) of the calcium-binding loop interact with Sr^2+^, compensating for the loss of water coordination. The average coordination distances with Sr^2+^ are a bit higher in the EF1 motif (by 0.1 Å) and almost same in the EF2 motif, compared to the average Ca^2+^-coordination distances.

### Barium complex

In CaM, Ba^2+^ has low binding affinity for EF-hand motifs compared to Ca^2+^[10] For EhCaBP1 also, Ba^2+^ has lower binding energy compared to Ca^2+^, even though the difference in binding energy is not very high, therefore it cannot replace the calcium at low concentrations. Hence, the EhCaBP1 was denatured and refolded in Ba^2+^ containing buffer as described above.

The overall structure of the Ba^2+^-EhCaBP1 complex, including the coordination geometry, is similar to that seen in the native structure, except for a couple of features (Figure 1) The coordination distances in the EF1 motif are on average 0.1 Å longer in Ba^2+^-EhCaBP1 than in the Ca^2+^-bound structure. This difference suggests that the EF-hand motif is flexible enough to accommodate metal ions of varied size (Figure 2D).

**Figure 3:**
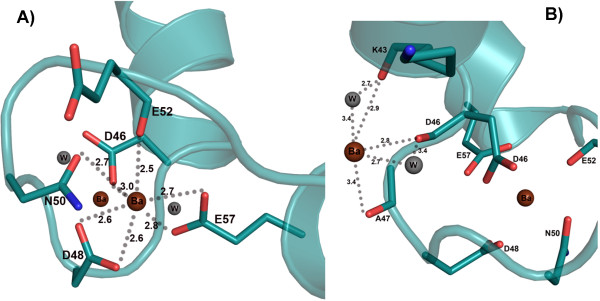
**Unusual binding of barium to the EF-hand 2 of EhCaBP1.** Barium has dual, partial occupancy to the EF hand 2 motif. (**A**) Barium coordinates with seven ligands from the protein to satisfy the coordination geometry. The coordination distances are shown. (**B**) Another barium ion in the close vicinity of EF-hand 2 motif is coordinated with the backbone oxygen atoms of K43, D46 and D48 residues of the protein and with two water molecules. The coordination distances between the barium ion and oxygen atoms range between 2.7 Ǻ and 3.4 Ǻ.

Moreover, a significant difference occurs in the EF2 motif, where two barium sites are observed. One barium is located at the same site as calcium The extra barium is in the close vicinity of EF-hand 2 loop, with partial occupancy and coordinated by residues K43, D46 and D48 (Figure 3). Both barium ions in the EF2 motif seem to be partially occupied, according to analyses of temperature factors and difference Fourier peaks. When assigned full occupancies, these bariums in the EF2 hand refined with high temperature factors compared to the barium ion bound in the EF1 hand loop as well as compared to other atoms in the EF2 hand. After reducing the occupancy to 0.5, the temperature factors were comparable to those of the barium atom in the EF1 motif. These two sites were confirmed by anomalous map and difference Fourier map up to 5σ before fitting barium in the electron density map. Before placing the heavy atoms, the fully occupied barium ion site had difference Fourier electron density at about 10σ and partially occupied barium ions has about 5σ level, clearly indicating the occupancy level. Similar binding pattern was also observed in EF-hand 2 of barium-soaked CaM crystal structure, but no barium was found to be bound to its other EF hand motifs [17].

### Anomalous signal analysis and verification of heavy atom binding

The barium complex shows a good anomalous signal of 0.1071, the signal for the lead complex is 0.0622, and the strontium complex data exhibits no anomalous signal at the respective wavelengths for which the data sets were collected. The calculated anomalous signals of the three data sets, the number of heavy atom binding sites in the proteins, as well as the f’ values of the heavy atoms all follow a similar trend. Since the Ba^2+^ is bound at three sites per molecule (one ion with full occupancy and two ions with partial occupancy) in the Ba^+2^-EhCaBP1 structure, its data showed the highest anomalous signal.

### Thermodynamics of EhCaBP1-Ca/Sr/Ba complex formation

**Figure 4:**
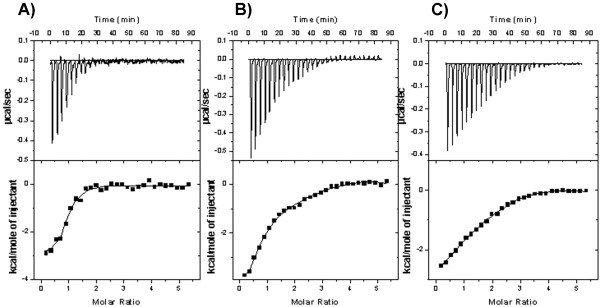
**ITC profile of the EhCaBP1-Ca (A), EhCaBP1-Sr (B), and EhCaBP1-Ba (C) associations at 25°C.** Upper panel– raw data for sequential 10 μL injections of 0.5 mM metals into 0.02 mM EhCaBP1 solution; Lower panel–the data points obtained by integration of the peaks in the upper panel, and plotted against the molar ratio (metal: protein). The squares are the experimental data and the solid line represents the best fit obtained from the data fitting by sequential mode. Further details are given in the materials and methods section.

The ITC data indicate that EhCaBP1 has four sequential binding sites for Ca^2+^ and Sr^2+^, whereas the protein has five sequential binding sites for Ba^2+^ (Figure 4), consistent with the trends observed in the crystal structures described above. (Note that throughout the manuscript, “sites 1, 2, 3, 4 and 5” only correspond to the order of occupied sites during the fitting of ITC data by sequential mode. These ITC identifiers of binding sites do not necessarily correspond to the names of the EF hand motifs depicted in the crystal structures).

It is well known that metals induce large conformational changes in calmodulin-like proteins, including EhCaBP1 [21]. Hence it is very difficult to conclude whether the obtained thermodynamic parameters are associated only with binding a metal ion or with synchronous conformational changes. But the accurate determination of the thermodynamic parameters of protein-ligand interaction incorporated with ligand-induced conformational alterations, different contributions in the change of free energy, enthalpy and entropy are helpful in understanding ligand binding and conformational changes in protein structure. The binding of ligand to protein would be expected to be enthalpically-driven in the absence of any additional conformational changes in the protein. Replacing water molecules with ligand will, however, influence the entropy.

**Figure 5:**
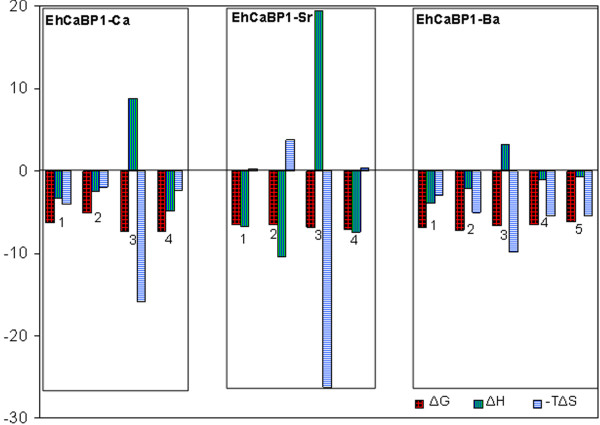
**Thermodynamic signatures for Ca**^**2+**^**, Sr**^**2+**^**and Ba**^**2+**^**associations to EhCaBP1.** The corresponding binding sites are in Roman capitals.

The change in free energy (ΔG) of the binding of metal to EhCaBP1 is negative for every case (Table 2), revealing that binding occurs spontaneously. Except for site 2 of Ca^2+^, it is observed that although the binding enthalpies and entropies of Ca^2+^, Sr^2+^ and Ba^2+^ to EhCaBP1 interaction differ a lot, their binding affinities (ΔG) remain effectively the same because changes in the binding enthalpy are compensated by changes in the binding entropy (Figure 5, Table 2). Consequently, enthalpically more favorable binding essentially results in greater entropic constraint, and thus in more unfavorable entropy. It is possible that a large increase in entropy derived from changes in water structure drives the entropically contributed interaction in the first site of Ca^2+^ as well as sites 2, 4 and 5 of Ba^2+^.

**Table 2:** Thermodynamic parameters of metal binding to EhCaBP1in 20 mM MOPS buffer pH 7.4 at 25°C obtained and calculated from ITC results

**Metal**	Site*	**K**_**a**_**(M**^**-1**^**)**	**ΔH(kcal.mol**^**-1**^**)**	**ΔS(Cal.mol**^**-1**^**.K**^**-1**^**)**	**ΔG(kcal.mol**^**-1**^**)**	**Dominating forces (inferred)**
Ca^2+^	1	2.56E5 ± 9.1E4	−3.33 ± 0.30	13.6	−7.38	H-bonding/hydrophobic interactions
	2	1.60E3 ± 9.0E2	−2.42 ± 0.28	6.54	−4.37	H-bonding/hydrophobic interactions
	3	2.42E5 ± 8.3E4	8.80 ± 0.36	53.2	−7.34	Hydrophobic interactions
	4	1.97E5 ± 8.6E4	−4.85 ± 0.94	7.94	−7.22	H-bonding/hydrophobic interactions
Sr^2+^	1	5.57E4 ± 9.6E3	−6.74 ± 0.51	−0.90	−6.47	H-bonding /conformational change
	2	6.44E4 ± 7.0E3	−10.35 ± 1.98	−12.7	−6.56	H-bonding /conformational change
	3	1.06E5 ± 1.5E4	19.42 ± 2.71	88.1	−6.83	Hydrophobic interactions
	4	1.49E5 ± 2.1E4	−7.44 ± 1.31	−1.28	−7.06	H-bonding /conformational change
Ba^2+^	1	1.02E5 ± 1.5E4	−3.84 ± 0.17	10.0	−6.82	H-bonding/hydrophobic interactions
	2	1.88E5 ± 2.0E4	−2.15 ± 0.39	16.9	−7.18	H-bonding/hydrophobic interactions
	3	7.15E4 ± 6.1E3	3.23 ± 1.02	33.1	−6.62	Hydrophobic interactions
	4	6.25E4 ± 9.3E3	−1.09 ± 2.14	18.3	−6.55	H-bonding/hydrophobic interactions
	5	3.16E4 ± 1.9E3	−0.69 ± 0.23	18.3	−6.14	H-bonding/hydrophobic interactions

*Sites 1, 2, 3, 4 and 5 only correspond to the order of occupied sites during the non-linear fitting of ITC data by sequential mode. These are not exchange digits for the loop of EF-I, EF-II *etc.*

The most favorable binding reactions are both enthalpy and entropy driven due to specific hydrogen-bond formation and van der Waals forces as well as hydrophobic interactions [22]. Hence, EhCaBP1-metal associations with Ca^2+^ and Ba^2+^ except their site 3 are favorable reactions. In site 3, for every metal (Ca^2+^, Sr^2+^ and Ba^2+^), the interaction is highly entropically favored (despite with positive enthalpy) (Figure 5). These parameters for site 3 may be explained by an opening of the lobe and exposure of hydrophobic pockets, which are target sites in signal transduction. The transformation from the *apo* to *metal bound* form of a protein, through rearrangement of electrostatic bonds where metals and charged amino acids interact with each other through electrostatic forces, is a determining factor for the protein’s plasticity. Therefore, the degree to which electrostatic interactions (side chains) stabilize the protein may be determined in its flexible region.

The interaction of the “extra” Ba^2+^ with the K43/D46/A47 oxygens in EF-2 as observed in the crystal structure (Figure 3B) is directed by a combination of both the coordination and electrostatic forces. This interaction could be the 5^th^ site according to ITC studies. In general, if ΔH ≈ 0, ΔS >0, and the opposite charges are less than 3.5 Å apart, the interaction is mainly governed by electrostatic force; Such is the case for the binding of Ba^2+^ to the 5^th^ site, where ΔH, ΔS and the distances are −0.7 kCal.mol^-1^, 18.3 Cal.mol^-1^.K^-1^ and 2–3 Å respectively.

The binding of Sr^2+^ to site 1, site 4, and especially site 2 are purely enthalpy driven (Table 2). A high binding enthalpy may also be used for the prediction that the binding of Sr^2+^ to the loop displaces the bridging water molecule. Hence, structural alterations at the binding site due to the binding event may contribute to this enthalpy. The largest –TΔS value for the binding of Sr^2+^ is to site 2 and should correspond to the opening of the binding pocket. The results for site 1 and 4 may be due to the binding to the heterogeneous native state structure, which may be ensembles of minimum energy conformations. Further, like other metals, only the binding of Sr^2+^ to site 3 is purely entropic (Table 2, Figure 5). Hence, enthalpic contributions dominate the association of Sr^2+^ with EhCaBP1.

## Discussion

We have successfully replaced the calcium of EhCaBP1 with the heavy metal ions Pb^2+^, Ba^2+^ and Sr^2+^, crystallized the complexes, and determined their structures. The difference Fourier electron density and anomalous signal confirms the presence of these heavy metal ions in place of calcium at the calcium binding loops in the respective crystal structures. The overall conformation and metal-coordination geometry of these complexes are quite similar to those of Ca^2+^-bound EhCaBP1, except for some relatively minor differences (Figure 1). This overall similarity provides a structural rationale for the ability of EhCaBP1 complexed with Pb^2+^, Sr^2+^ or Ba^2+^ to bind and to activate some of the biological targets of Ca^2+^- EhCaBP1.

Lead has been reported several times to be the most effective heavy metal for replacing calcium in the CaM activation cascade [8,9]. No significant structural rearrangements occur upon replacement of calcium with lead, and the coordination geometry is similar [18]. As a result of lead’s high binding affinity, it is tightly bound to the EF hand motif even at relatively low concentrations, unlike Ca^2+^, thus providing a structural reason for the reported constitutively active state of Pb^2+^ -CaM [10,11]. It is probable that lead has similar effects on EhCaBP1. Since lead has a relatively high binding affinity for the calcium binding site and can replace calcium very easily in EhCaBP1 as well. It may very well also result in the aberrant activation of this (EhCaBP1) protein’s downstream signaling pathways.

Although the complexes of EhCaBP1 with the various metal ions are similar to one another, the binding of Sr^2+^ and Ba^2+^ to the protein show a couple of special features. It appears that the EhCaBP1-Sr association is of a pre-formed nature since all seven coordinating atoms are from the protein itself (Figure 2); in contrast, in EhCaBP1-Ca and EhCaBP1-Ba, only six coordinating atoms are supplied by the protein and the remaining one is from water. Hence, among the three heavy metal ions, it can be concluded that Sr^2+^ shows the most specific interactions with EF-hand motifs. In the crystal structures of EhCaBP1, for each of the EF-I and EF-II binding sites for Ca^2+^, and for the EF-I binding site for Ba^2+^, the metal coordinates one water molecule (Figure 2). In the EF2 motif, Ba^2+^ binds with partial occupancy to two adjacent binding sites, and, in one of these sites, coordinates with several water molecules (Figure 3). The ITC data of the association of EhCaBP1 with Ca^2+^ and Ba^2+^ show a positive change in entropy in all of the sites (Table 2). In contrast, in EhCaBP1-Sr, where both the EF-I and EF-II binding sites are devoid of water, the change in entropy is positive for only one site (site 3) and negative for the others (site 1, 2 and 4). This result suggests that if there is ligated water in an EhCaBP1-metal complex, the change in entropy will be positive. Therefore, in EhCaBP1-Sr, the only site with positive entropy of binding, i.e. site 3, must be involved in opening of hydrophobic pocket as discussed above (Figure 5). These findings also imply that the unusual thermodynamic signatures in EhCaBP1-Sr (Table 2) are just a result of the absence of any ligated water molecule.

It has been already reported that in the different signal transducers, calmodulin (CaM) and troponin C (TnC), calcium-induced conformational alterations resulting in exposure of hydrophobic target sites are mainly due to motions of secondary structural units [23]. The gross structural morphology, secondary structural contents, and target binding of EhCaBP1 are related to those of vertebrate CaM [24]. According to this, upon binding a metal (*apo* → *holo*), the molecule changes conformation from a closed to an open state conformation, with many H-bonds from waters to side chains and backbone being rearranged, as is observed in CaM and also opening of hydrophobic core. Upon metal interaction in the first site, the most compact loop is formed by the rearrangement of van der Waals and ion pair contacts. Consequently the energetic barriers for the formation of another binding loop will be lowered, and the loop will properly position binding residues. The same pattern could then be sequentially transmitted in a cooperative manner to the next binding site.

## Conclusion

Here, we have reported for the first time, the structural analysis of any calmodulin like protein, EhCaBP1 in complex with strontium. The Sr^2+^ -coordination in the EF hand motifs is similar to that of the native Ca^2+^ bound structure, except for the lack of water coordination. Sr^2+^ coordination seems to be a pre-formed in nature since all seven coordinating atoms are from the protein itself, which also correlates with entropy contributions in Sr^2+^ binding, in contrast, in EhCaBP1-Ca and EhCaBP1-Ba, only six coordinating atoms are supplied by the protein and the remaining one is from water. Our work has also provided the first successful example of replacing Ca^2+^ with Ba^2+^ in the EF1 motif of a calcium binding protein. Previously such information was restricted to the EF2 motif in CaM [17], and here as in our results for EhCaBP1 there are two bariums ions bound to EF2 motif. This is first time, any calcium binding protein structure is reported with four different heavy atoms, albeight with low resolution.

The crystallographic and ITC results clearly indicate that EhCaBP1 is also capable of binding and being activated by metal ions other than Ca^2+^, providing a better understanding of the activation mechanisms of EhCaBP1 by different heavy metals, and also give better insight into the flexibility of the calcium-binding loops of EhCaBP1. These results can be also extended to all EF hand containing proteins like Calmodulin. These results help us to explain the physiological basis for toxicity of these heavy metals by providing structural and biochemical insights into how these metal ions can effectively substitute for Ca^2+^ in several signaling pathways which are central to several regulatory processes.

## Methods

### Preparation of EhCaBP1-Pb complex, crystallization and data collection

EhCaBP1 was over-expressed and purified as previously described [19] and concentrated to 20 to 25 mg/ml. Since Pb^2+^ can easily replace Ca^2+^ from the calcium-binding loops of CaM, [10,11,18] Pb(NO3)_2_ at an initial concentration of 1 M was directly added to the purified EhCaBP1 to bring the final concentration of Pb^2+^ to 5 mM. Upon addition of Pb^2+^, the solution became turbid and protein precipitated. The solution was partially clarified upon addition of 1 M sodium acetate pH 4.0 to a final concentration of 100 mM. The EhCaBP1-Pb complex was centrifuged at 12,000 rpm to remove any precipitate, and the supernatant was used for crystallization.

Hanging-drop vapor-diffusion crystallization trials were carried out under conditions similar to that used for crystallization of the native protein [19]. Crystals suitable for diffraction were obtained by mixing 5 μl of EhCaBP1-Pb complex and 2 μl of reservoir solution containing 60–65% MPD in 50 mM sodium acetate pH 3.6. Rod shaped crystals (250 × 250 × 150 μm) appeared after 7–10 days of equilibration (Figure 1A).

X-ray diffraction experiments were done at 100 K with EhCaBP1-Pb crystals mounted on cryoloops in mother liquor and flash frozen in liquid nitrogen. These crystals diffracted to a resolution of 3.0 Å with an in-house Bruker Microstar rotating anode generator and a MAR345 image plate detector (Advanced Instrumentation Research Facility, JNU). The data sets were indexed, processed and scaled using the program Automar [25]. The crystals belong to the space group P6_3_ (Table 3) with two molecules per asymmetric unit, similar to that of the native structure [19] (Figure 1).

**Table 3:** Crystallographic data- statistics

**Data set**	**EhCaBP1-Pb**	**EhCaBP1-Sr**	**EhCaBP1-Ba**
**Crystallographic Data**			
X-ray Source	MicroStar	Elettra	MicroStar
Wavelength (Å)	1.5418	1.0	1.5418
Space group	P6_3_	P6_3_	P6_3_
Unit-cell parameters (Å)	*a =* 95.264,	*a =* 95.429,	*a =* 95.210,
	b = 95.264,	b = 95.429,	b = 95.210,
	c = 64.597	c = 63.973	c = 62.986
Resolution range (Å)	28.08-3.0	30-3.0	50-3.2
R_sym_ (%)	4.66 (36.4)	6.80 (45.4)	6.70 (62.8)
Completeness (%)	99.8 (100)	94.9 (96.8)	99.6 (98.7)
Observations (*N*)	24659	26059	28321
Unique observations (*N*)	6763	6442	5485
Redundancy	8.8 (8.1)	4.0 (4.0)	5.2 (4.4)
Average *I/s (I)*	15.2 (1.9)	18.81 (2.8)	15.4 (1.8)
Crystal mosaicity (º)	0.5	0.5	1.0
**Refinement**			
Resolution (Å)	28.08-3.0	30-3.0	50-3.2
R-factor (%)	24.24	25.23	24.70
Free R-factor (%)	28.67	27.99	31.40
Mean B-factor(Å^2^)	71.80	95.18	122.09
Atoms (*N*) Protein/HA/ACT/water	996/4/2/45	1000/4/0/27	966/6/0/21
**RMS deviations**			
Bonds (Å)	0.009	0.008	0.010
Bond angles (°)	1.7	1.2	1.3
Cross validated error	0.47	0.50	0.49

HA = Heavy Atom, ACT = Acetate.

Values in parentheses are for the highest resolution shell.

Free R-factors were calculated with a subset of randomly selected reflections (10.7% for EhCaBP1-Pb, 9.4% for EhCaBP1-Sr and 4.4% for EhCaBP1-Ba complex).

### Preparation of EhCaBP1-Ba complex, crystallization and data collection

High concentrations of Ba^2+^ is required for the activation of CaM compared to Pb^2+^[26] Since Ba^2+^ has lower binding affinity for EF-hand motifs than does Ca^2+^, it cannot simply replace Ca^2+^. To replace all of the bound calcium ions from the calcium binding loops, EhCaBP1 was unfolded and calcium ions were removed by treatment with 8 M urea and 5 mM EGTA, The protein was dialyzed against 10 mM barium chloride in Tris buffer (pH 7.5) several times to remove urea and EGTA and then the protein was refolded in presence of excess barium ions. The EhCaBP1-Ba complex was then concentrated to 20–25 mg/ml, and crystallized by the hanging-drop vapor-diffusion method using conditions similar to that used for the native protein. Crystals suitable for diffraction were obtained by mixing 5 μl of EhCaBP1-Ba complex and 5 μl of the reservoir solution (60–65% MPD, 50 mM sodium acetate pH 3.8). Rod shaped crystals with oiling appeared after 15–20 days of equilibration.

The X-ray diffraction experiments were done at 100 K with EhCaBP1-Ba crystals mounted on cryoloops in mother liquor and flash frozen in liquid nitrogen stream. These crystals diffracted to a resolution of 3.2 Å using an in-house rotating anode generator (Advanced Instrumentation Research Facility, JNU). The data sets were indexed, processed and scaled using Automar [25]. They belong to the space group P6_3_ (Table 3) with two molecules per asymmetric unit, similar to that of the native structure [19].

### Preparation of EhCaBP1-Sr complex, crystallization and data collection

The EhCaBP1-Sr complex was prepared and crystallized following protocols similar to those used for the EhCaBP1-Ba complex. Crystals suitable for diffraction were obtained by mixing 3 μl of the EhCaBP1-Sr complex and 3 μl of the reservoir solution (60–65% MPD, 50 mM sodium acetate pH 3.6). Hexagonal crystals appeared after 15–20 days of equilibration. The X-ray diffraction experiments were done at 100 K with EhCaBP1-Sr crystals mounted on cryoloops in mother liquor and flash frozen in liquid nitrogen stream. These crystals diffracted to a resolution of 3.0 Å at beamline BL 5.2 R, XRD1, Elettra Synchrotron Source, Trieste, Italy. The data sets were indexed, processed and scaled with HKL2000 program [27]. They, too, belong to space group P6_3_ with two molecules per asymmetric unit.

### Structure determination

All three complex structures were solved by molecular replacement with the program Phaser [28] using the unliganded native structure of EhCaBP1 (2NXQ) as the search model. Iterative model building by the COOT graphics package [29] combined with Phenix.refine and Refmac5 for refinement was carried out to determine the EhCaBP1-Ba and EhCaBP1-Pb complex structures. CNS refinement with conjugate-gradient minimization and bulk solvent correction [30] was used for both the EhCaBP1-Sr and complex, whose structures are more ordered than the EhCaBP1-Ba complex structure (Table 3).

Calcium binding sites showed significant density peaks in the difference fourier maps of all the three heavy atom complexed structures compared to calcium bound structure. The peak in the barium bound difference map was strongest, at 6σ, Lead at 4σ, whereas that of the strontium bound structure was at a 3σ level, following the trend of the molecular weights of heavy atoms bound compared to calcium. The best R-factors were not achieved until the replacement of Ca^2+^ with the respective heavy atoms. The final models refined well, fitting most of the electron density (for the N-terminal domain), and yielded crystallographic R_factor_ and R_free_ values that are within the range of average values for structures refined at the given resolutions [31]. The 2Fo-Fc electron density maps for the heavy atoms were so strong that the density was even seen at 10σ for Barium, 6σ for lead and 7σ for strontium ( Additional file 1: Figure S1). The temperature factors for Lead were very high compared to temperature factor from Wilson plot. The occupancy for lead was adjusted to 0.5 to get comparable temperature factors. Generally, the protein was urea denatured followed by refolding in presence of the respective heavy metals containing buffers except for lead. Lead has more affinity than calcium, supposed to occupy the calcium-binding sites more easily. This may be the reason why the lead ions have half occupancies. Despite acceptable refinement statistics, electron density for the C-terminal half of the molecule was absent, as occurs in the native structure. In the final stages of refinement, water molecules were added manually where the Fo-Fc electron density exceeded 3.0 σ and when justified by hydrogen bonds. Only a few water molecules met these criteria.

### Protein data bank accession codes

Coordinates and structure factors have been deposited in PDB with accession code 3PX1 for EhCaBP1-Sr, 3QJK for EhCaBP1-Pb and 3ULG for EhCaBP1-Ba complexes.

### Anomalous signal analysis

To confirm the presence of heavy atoms at the EF-hand motifs, the anomalous signal was checked. Barium and lead atoms have f” of about 9 electrons at Cu-Kα wavelength of 1.54 Å, where as strontium has F” of 1.845 electrons at 1.2 Å. The anomalous signal was calculated using phenix.xtriage [32] for all data sets (Table 1).

### Isothermal titration calorimetry (ITC)

All solutions were demetallized by a Chelex-100 resin (BioRad). The protein concentration was determined by using ε = 5120 M^-1^ cm^-1^ at 280 nm [33]. The calorimetric measurements were carried out using a titration calorimeter from Microcal (Northampton, MA) at 25°C. The solutions of EhCaBP1 and metals were prepared in the same buffer. The 0.02 mM EhCaBP1 solution in the 1.44 ml sample cell was titrated with 0.5 mM calcium chloride (Ca^2+^), strontium chloride (Sr^2+^) and barium chloride (Ba^2+^) dissolved in the same buffer using a 288 μl automatic rotating syringe stirring at 307 rpm. Titration experiments consisted of 28 injections of 10 μl each of duration 20s with 2 s filter period and 180 s spacing between each injection. The analog input range was +/− 1.25 V and the reference power was set at 20 μcal s^-1^. The heat associated with each injection was observed, as a peak that corresponded to the power required to keep the sample and reference cells at identical temperatures. Control experiments were performed by titrating metals into the same buffer to obtain the heats of ligand dilution. The net enthalpy for each EhCaBP1-metal association was determined by subtraction of the component heats of dilution from each injection heat pulse. Integration with respect to time of the heats produced per serial injection of metal yielded the corresponding binding isotherm. The binding isotherms were fitted for a model of sequential binding sites by using Origin 7.0 provided with the MicroCal instrument (Table 2). The titration with Pb^2+^ could not be done, as the lead salts were insoluble at physiological pH 7.4. The details of curve fitting and determination of number of binding sites for each heavy atom are described in Additional file 1.

## Abbreviations

TnC: Troponin C; CaM: Calmodulin; MPD: 2-methyl-2, 4-pentanediol; EhCaBP1: *Entamoeba histolytica* calcium binding protein 1; PDB: Protein data bank.

## Competing interests

The authors declare that they have no competing interests.

## Authors’ contributions

SK and SK purified the protein, crystallized and determined the structures with different heavy metals, analyzed and submitted the coordinates. EA and RHK did ITC experiments, analyzed the data and presented. SG planned, conceptualized, written the manuscript, raised the funds, also discussed and helped SK and SK in every step, where ever needed. All authors read and approved the final manuscript.

## Supplementary Material

Additional file 1 Electron density for heavy metals and Details of ITC experiments. Click here for file

## References

[B1] Cline HT Witte S Jones KW Low lead levels stunt neuronal growth in a reversible manner Proc Natl Acad Sci U S A 1996 93 9915 9920 10.1073/pnas.93.18.9915 8790431 PMC38529

[B2] Lidsky TI Schneider JS Lead neurotoxicity in children: basic mechanisms and clinical correlates Brain 2003 126 5 19 10.1093/brain/awg014 12477693

[B3] Apostoli P Bellini A Porru S Bisanti L The effect of lead on male fertility: a time to pregnancy (TTP) study Am J Ind Med 2000 38 310 315 10.1002/1097-0274(200009)38:3<310::AID-AJIM10>3.0.CO;2-9 10940969

[B4] Hernandez-Ochoa I Garcia-Vargas G Lopez-Carrill L Rubio-Andrade M Moran-Martinez J Cebrian ME Quintanilla-Vega B Low lead environmental exposure alters semen quality and sperm chromatin condensation in northern Mexico Reprod Toxicol 2005 20 221 228 10.1016/j.reprotox.2005.01.007 15907657

[B5] Moore MR Goldberg A Yeung-Laiwah AA Lead effects on the heme biosynthetic pathway. Relationship to toxicity Ann N Y Acad Sci 1987 514 191 203 10.1111/j.1749-6632.1987.tb48774.x 3442384

[B6] Atchison WD Effects of toxic environmental contaminants on voltage-gated calcium channel function: from past to present J Bioenerg Biomembr 2003 35 507 532 15000519 10.1023/b:jobb.0000008023.11211.13

[B7] Chao SH Bu CH Cheung WY Activation of troponin C by Cd2+ and Pb2+ Arch Toxicol 1990 64 490 496 10.1007/BF01977632 2148867

[B8] Aramini JM Hiraoki T Grace MR Swaddle TW Chiancone E Vogel HJ NMR and stopped-flow studies of metal ion binding to alpha-lactalbumins Biochim Biophys Acta 1996 1293 72 82 10.1016/0167-4838(95)00223-5 8652630

[B9] Fullmer CS Edelstein S Wasserman RH Lead-binding properties of intestinal calcium-binding proteins J Biol Chem 1985 260 6816 6819 3997849

[B10] Chao SH Bu CH Cheung WY Stimulation of myosin light-chain kinase by Cd2+ and Pb2+ Arch Toxicol 1995 69 197 203 10.1007/s002040050158 7717877

[B11] Habermann E Crowell K Janicki P Lead and other metals can substitute for Ca2+ in calmodulin Arch Toxicol 1983 54 61 70 10.1007/BF00277816 6314931

[B12] Forsen S Thulin E Lilja H 113Cd NMR in the study of calcium binding proteins: troponin C FEBS Lett 1979 104 123 126 10.1016/0014-5793(79)81097-2 477971

[B13] Pidcock E Moore GR Structural characteristics of protein binding sites for calcium and lanthanide ions J Biol Inorg Chem 2001 6 479 489 10.1007/s007750100214 11472012

[B14] Wang CL Leavis PC Gergely J Kinetic studies show that Ca2+ and Tb3+ have different binding preferences toward the four Ca2 + −binding sites of calmodulin Biochemistry 1984 23 6410 6415 10.1021/bi00321a020 6529556

[B15] Yang W Wilkins AL Ye Y Liu ZR Li SY Urbauer JL Hellinga HW Kearney A van der Merwe PA Yang JJ Design of a calcium-binding protein with desired structure in a cell adhesion molecule J Am Chem Soc 2005 127 2085 2093 10.1021/ja0431307 15713084

[B16] Vyas MN Jacobson BL Quiocho FA The calcium-binding site in the galactose chemoreceptor protein. Crystallographic and metal-binding studies J Biol Chem 1989 264 20817 20821 2684986

[B17] Kursula P Majava V A structural insight into lead neurotoxicity and calmodulin activation by heavy metals Acta Crystallogr Sect F Struct Biol Cryst Commun 2007 63 653 656 10.1107/S1744309107034525 PMC2335165 17671360

[B18] Wilson MA Brunger AT Domain flexibility in the 1.75 A resolution structure of Pb2+−calmodulin Acta Crystallogr D: Biol Crystallogr 2003 59 1782 1792 10.1107/S0907444903016846 14501118

[B19] Kumar S Padhan N Alam N Gourinath S Crystal structure of calcium binding protein-1 from Entamoeba histolytica: a novel arrangment of EF hand motifs Proteins 2007 68 990 998 10.1002/prot.21455 17554780

[B20] Kumar S Ahmad E Mansuri MS Kumar S Jain R Khan RH Gourinath S Crystal structure and trimer-monomer transition of N-terminal domain of EhCaBP1 from Entamoeba histolytica Biophys J 2010 98 2933 2942 10.1016/j.bpj.2010.03.048 20550906 PMC2884261

[B21] Gopal B Swaminathan CP Bhattacharya S Bhattacharya A Murthy MR Surolia A Thermodynamics of metal ion binding and denaturation of a calcium binding protein from Entamoeba histolytica Biochemistry 1997 36 10910 10916 10.1021/bi9702546 9283081

[B22] Velazquez-Campoy A Todd MJ Freire E HIV-1 protease inhibitors: enthalpic versus entropic optimization of the binding affinity Biochemistry 2000 39 2201 2207 10.1021/bi992399d 10694385

[B23] Strynadka NC Cherney M Sielecki AR Li MX Smillie LB James MN Structural details of a calcium-induced molecular switch: X-ray crystallographic analysis of the calcium-saturated N-terminal domain of troponin C at 1.75 A resolution J Mol Biol 1997 27 238 255 9367759 10.1006/jmbi.1997.1257

[B24] Jain R Kumar S Gourinath S Bhattacharya S Bhattacharya A N- and C-terminal domains of the calcium binding protein EhCaBP1 of the parasite Entamoeba histolytica display distinct functions PLoS One 2009 4 e5269 10.1371/journal.pone.0005269 19384409 PMC2668073

[B25] Bartels KS Klein C The AUTOMAR Manual, v.1.4 2003 Norderstedt, Germany: MAR Research GmbH

[B26] Yamazaki J Urushidani T Nagao T Barium activates rat cerebellar nitric oxide synthase Jpn J Pharmacol 1996 70 351 354 10.1254/jjp.70.351 8774764

[B27] Otwinowski O Minor W Processing of X-ray diffraction data collected in oscillation mode Meth Enzymol 1997 276 307 326 10.1016/S0076-6879(97)76066-X 27754618

[B28] Storoni LC McCoy AJ Read RJ Likelihood-enhanced fast rotation functions Acta Crystallogr D: Biol Crystallogr 2004 60 432 438 10.1107/S0907444903028956 14993666

[B29] Emsley P Cowtan K Coot: model-building tools for molecular graphics Acta Crystallogr D: Biol Crystallogr 2004 60 2126 2132 10.1107/S0907444904019158 15572765

[B30] Brunger AT Adams PD Clore GM DeLano WL Gros P Grosse-Kunstleve RW Jiang JS Kuszewski J Nilges M Pannu NS Read RJ Rice LM Simonson T Warren GL Crystallography & NMR system: A new software suite for macromolecular structure determination Acta Crystallogr D: Biol Crystallogr 1998 54 905 921 10.1107/S0907444998003254 9757107

[B31] Kleywegt GJ Brunger AT Checking your imagination: applications of the free R value Structure 1996 4 897 904 10.1016/S0969-2126(96)00097-4 8805582

[B32] Zwart PH Anomalous signal indicators in protein crystallography Acta Crystallogr D: Biol Crystallogr 2005 61 1437 1448 10.1107/S0907444905023589 16239720

[B33] Gopal B Krishna Rao JV Thomas CJ Bhattacharya A Bhattacharya S Murthy MR Surolia A Induction of a spectroscopically defined transition by guanidinium hydrochloride on a recombinant calcium binding protein from Entamoeba histolytica FEBS Lett 1998 441 71 76 10.1016/S0014-5793(98)01513-0 9877168

