# Supplementary material for: Flexibility of EF-hand motifs: structural and thermodynamic studies of Calcium Binding Protein-1 from Entamoeba histolytica with Pb2+, Ba2+, and Sr2+
Source: BMC Biophys. 2012 Aug 20;5:15. doi: 10.1186/2046-1682-5-15 (PMC3483242; doi:10.1186/2046-1682-5-15)
Supplement: Additional file 1 — Electron density for heavy metals and Details of ITC experiments. [file 2046-1682-5-15-S1.docx]

**Supplementary Figure 1.** The metal binding site with EF hand motif of EhCaBP1 in complex with different heavy atoms. A) EF1 motif in complex with Barium superimposed with electron density (2Fo-Fc) at 10σ cut off. B) EF2 motif in complex with Barium superimposed with electron density at 5σ cut off. C) EF1 motif in complex with lead superimposed with electron density at 6σ cut off. D) EF1 motif in complex with strontium superimposed with electron density at 7σ cut off.

**Isothermal titration calorimetry (ITC):**

For sequential binding, the binding constants are defined relative to the progress of saturation, such as

K_1_ = [PM]/[P][M]

K_2_ = [PM_2_]/[PM][M]

K_3_ = [PM_3_]/[PM_2_][M]

and so on up to total number of sequential binding sites where M denotes metal ion and P denotes the protein molecule [Kishore]. The heat content Q after any *i*^th^ injection is then expressed as:

**Q = P_t_V_o_[F_1_ΔH_1_ + F_2_(ΔH_1_ + ΔH_2_) + ··· + F_n_(ΔH_1_ + ΔH_2_ + ΔH_3_ + ··· +ΔH_n_)]**

where F*_i_* is the fraction of total macromolecule having *i* bound ligands and V_o_ is the active cell volume, whereas ΔH_1_, ΔH_2_ and ΔH_3_ correspond to the enthalpy change associated with the binding of the first, second and third metal to the protein. The pertinent calculated heat effect (ΔQ) for the ith injection is:

**ΔQ = Q(i) + dV_i_/V_o_ [(Q(i) + Q(i – 1))/2] – Q(i – 1)**

which is then used in the Marquardt minimization algorithm [Bevington, 1969] to obtain best fitting values until constant *χ*2 values were achieved for the determination of the association constants (K_a_ values), stoichiometry (n) and enthalpy change (ΔH). Other thermodynamic parameters such as change in free energy (ΔG) and change in entropy (ΔS) were obtained from:

ΔG = -RTlnK_a_

and second law of thermodynamics:

ΔG = ΔH-TΔS

***Determination of number of Ca^+2^, Sr^+2^ and Ba^+2^ binding sites in EhCaBP1:***

To determine the number of metal binding sites on the EhCaBP1 the goodness of fit was determined by calculation of *χ*^2^ from:

where *N* is the number of data points, *y_i_* the actual value, *f*(*x_i_*) the theoretical value and *σ_i_* is the measurement error. The data fits were acceptable in each case since the *χ*^2^ values were less than the critical values for the appropriate degree of freedom [Frazier, 2006]. In each case of EhCaBP1-metal complex formation the data fit in single, double and sequential binding modes were analyzed to get the lowest value of *χ*^2^. From Supplementary figure 1 it is clear that in case of EhCaBP1-Ca and EhCaBP1-Sr, four sequential binding sites are most appropriate because of their lowest *χ*^2^ values whereas there are five sites for EhCaBP1-Ba.

**Supplementary Figure 2.** The ‘χ^2^/DoF’ values obtained after the fitting the ITC data to different binding site models (1 to 6 sites) of EhCaBP1- with different metal interactions.
